# Supplementary material for: Synergy between Mecillinam and Ceftazidime/Avibactam or Avibactam against Multi-Drug-Resistant Carbapenemase-Producing Escherichia coli and Klebsiella pneumoniae
Source: Antibiotics (Basel). 2022 Sep 20;11(10):1280. doi: 10.3390/antibiotics11101280 (PMC9599007; doi:10.3390/antibiotics11101280)
Supplement: Supplementary file 1 [file antibiotics-11-01280-s001.zip › antibiotics-1895731-supplementary.pdf]

Supplementary Material

Purification of E. coli MG1655 membrane proteins

An overnight MHII broth culture of *E. coli* MG1655 cells was diluted 10x in 500 mL fresh MHII broth and grown at 37°C until reaching $OD_{600} \approx 1.0$. The harvest of cells was performed by centrifugation at 6000xg for 10 min at 4°C, followed by 2x cell washing with phosphate buffer solution (PBS). Cells, dissolved in PBS, were lysed by a Branson Ultrasonic Probe Sonicator at amplitude = 10%, Pulse ON = 30 sec, Pulse OFF = 30 sec for 2x5 min. The lysate was centrifuged at 16,000xg for 15 min to remove cells, which were not lysed. Membrane proteins were collected from supernatant by ultracentrifugation at 150,000xg for 1 h at 4°C. After centrifugation, the supernatant was discarded, and the protein pellet was suspended in 500 µL fresh cold PBS. Membrane protein solutions were stored at -20°C. A Bradford analysis (BIO-RAD, USA) was performed to determine protein concentrations.

Table S1. β-lactamase genes present in the 18 clinical isolates of *Escherichia coli* and *Klebsiella pneumoniae.* EC: *Escherichia coli,* KP: *Klebsiella pneumoniae,* KPC: *Klebsiella pneumoniae* carbapenmase, IMP: Imipenem metallo-beta-lactamase, NDM: New Dehli metallo-beta-lactamase, OXA: oxacillinase, VIM: Verona integron-encoded metallo-beta-lactamase, CTX-M: Extended Spectrum beta-lactamase, CMY: AmpC beta-lactamase, SHV: beta-lactamase, TEM: beta-lactamase.

Strain	Carbapenemase	Other beta-lactamases					
**KP1**	KPC-2	TEM-1A-like	SHV-11-like	OXA-9-like			
**KP2**	KPC-3	TEM-1B	SHV-11	OXA-1, OXA-9-like			
**KP3**	KPC-3		SHV-11-like				
**KP4**	KPC-3		SHV-1				
**KP5**	KPC-3	TEM-1B-like	SHV-28	OXA-1, OXA-9-like	CTX-M-15		
**KP6**	NDM-1	TEM-1B	SHV-11-like	OXA-1	CTX-M-15	CMY-6	DHA-1-like
**KP7**	NDM-1, OXA-48	TEM-1B	SHV-12	OXA-1	CTX-M-15		
**KP8**	NDM-5	TEM-1B	SHV-1	OXA-1	CTX-M-15		
**KP9**	NDM-7, OXA-181	TEM-1B	SHV-11-like		CTX-M-15	CMY-6	
**KP10**	OXA-232	TEM-1B	SHV-1		CTX-M-15		
**KP11**	OXA-436	TEM-1B	SHV-1-like	OXA-10			
**KP12**	IMP-1		SHV-26				
**ATCC 13883**							
**EC2**	NDM-1			OXA-1		CMY-6-like	DHA-1-like
**EC3**	NDM-5			OXA-1	CTX-M-15	CMY-42	
**EC4**	NDM-5, OXA-181	TEM-1B		OXA-1	CTX-M-15	CMY-2	
**EC5**	NDM-7	TEM-1b			-CTX-M-27		
**EC6**	OXA-244	TEM-1B			CTX-M-14b		
**EC7**	OXA-48	TEM-1B-like					

ATCC
25922

Time-kill assays

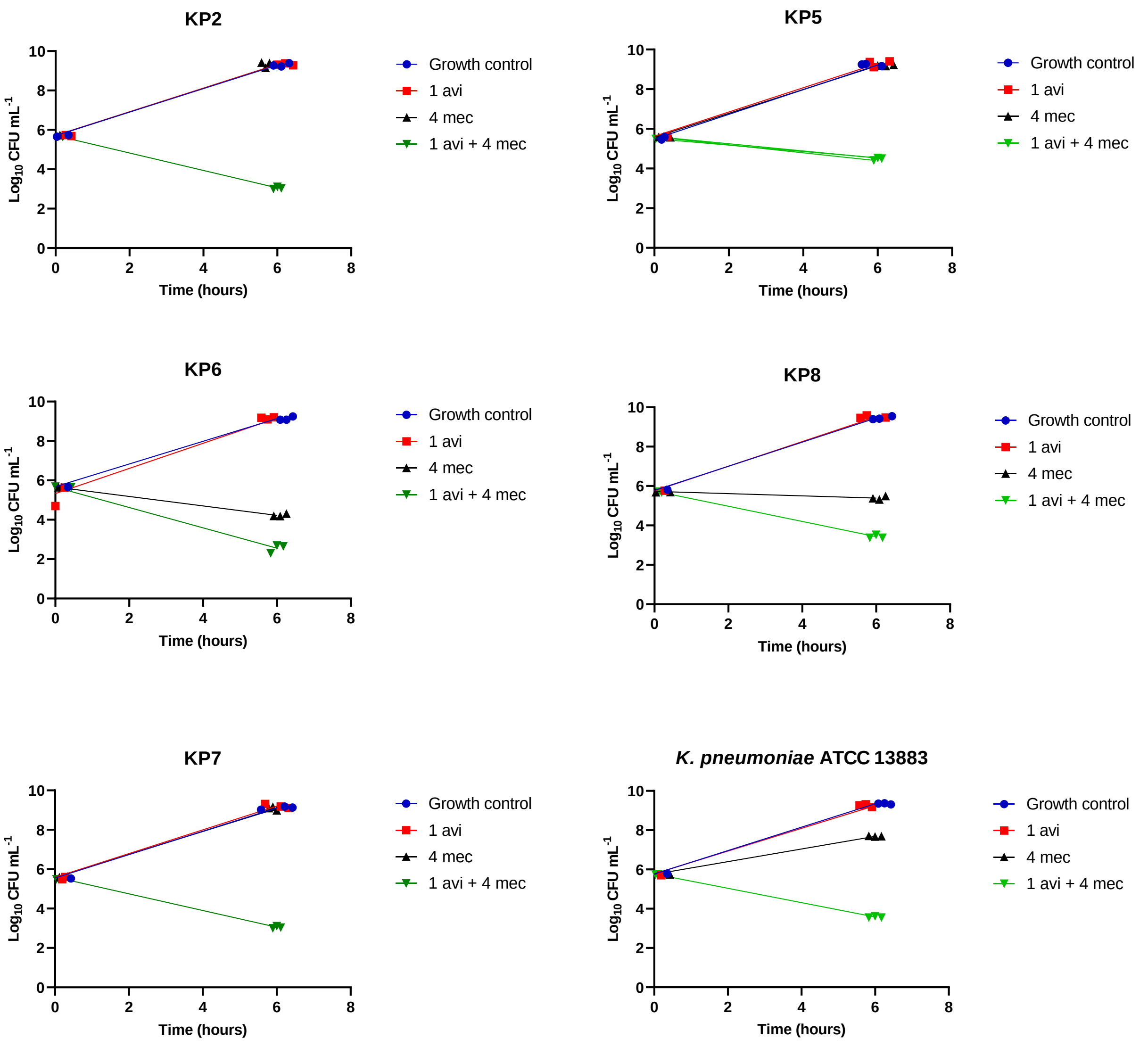

Figure S1. Time kill curves of five different *K. pneumoniae* isolates, KP2, KP5, KP6, KP7, KP8, and *K. pneumoniae* ATCC 13883 treated with 1 µg/ml avibactam, 4 µg/ml mecillinam, and 4 µg/ml mecillinam combined with 1 µg/ml avibactam for 6 h. Data are presented as log CFU ml^{-1}. AVI = avibactam, MEC = mecillinam.

Table S2. Mean log change after time kill experiments for five different *K. pneumoniae* isolates, KP2, KP5, KP6, KP7, KP8, and *K. pneumoniae* ATCC 13883 treated with 1 µg/ml avibactam, 4 µg/ml mecillinam, and 4 µg/ml mecillinam combined with 1 µg/ml avibactam for 6 h. Data are presented as log CFU ml^{-1}. AVI = avibactam, MEC = mecillinam.

		Mean log change (6 h)		
		Treatment group		
**Strain**	**Growth control**	**4 MEC (µg/ml)**	**1 AVI (µg/ml)**	**4 MEC + 1 AVI (µg/ml)**
**KP2**	3.80	3.61	3.61	-2.66
**KP5**	3.74	3.61	3.67	-1.11
**KP6**	3.45	-1.44	3.83	-3.1
**KP7**	3.56	3.49	3.61	-2.54
**KP8**	3.68	-0.34	3.76	-2.31
**ATCC 13883**	3.57	1.94	3.48	-2.2

KP: Klebsiella pneumoniae.

Phase contrast microscopy

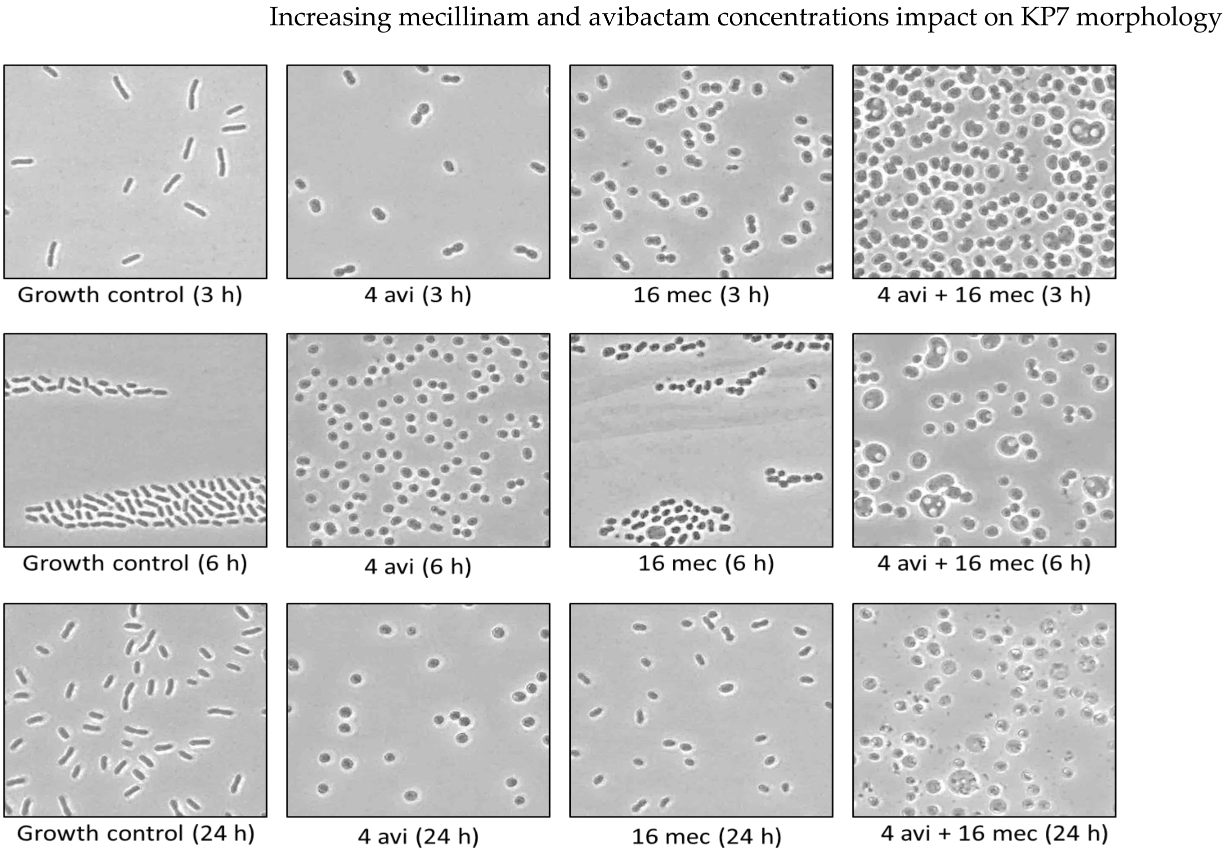

Figure S2. Phase contrast microscopy (100x magnification) of KP7 cells in four treatment groups, including growth control, 4 μg/ml avibactam (4 AVI), 16 μg/ml mecillinam (16 MEC), and 4 μg/ml avibactam combined with 16 μg/ml mecillinam (4 AVI + 16 MEC). Images are from time point 3, 6, and 24 h. .

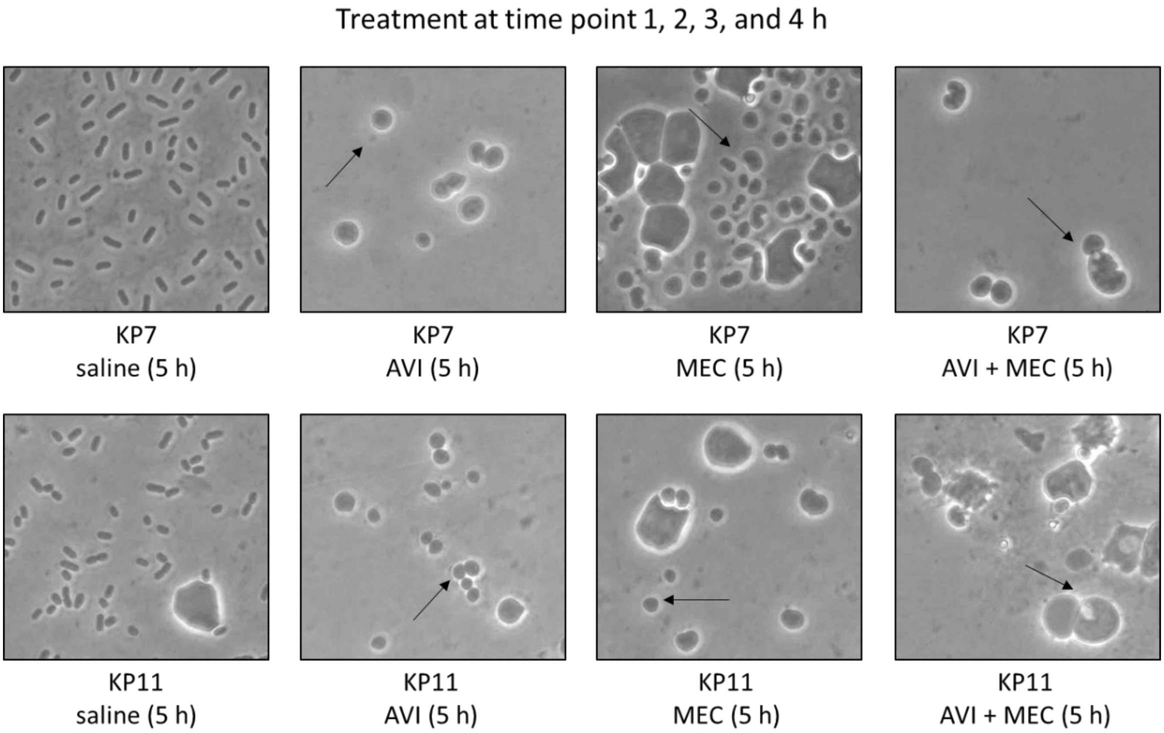

Figure S3. Phase contrast microscopy (100x magnification) of KP7 and KP11 cells *ex vivo* after 5 h treatment with saline, 100 mg kg^{-1} avibactam (AVI), 200 mg kg^{-1} mecillinam (MEC), and 100 mg kg^{-1} avibactam combined with 200 mg kg^{-1} mecillinam. Treatment at time point 1, 2, 3, and 4 h. Arrow, bacterial cell. .

Confocal laser scanning microscopy

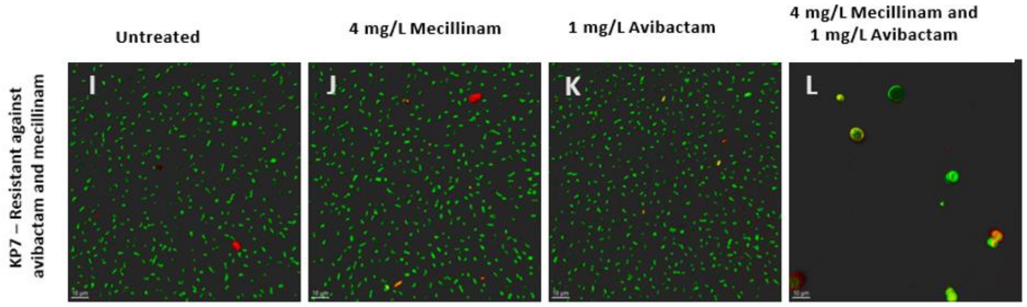

Figure S4. 3D projection of confocal laser scanning microscopy of cultures treated either 4 μg mL^{-1} mecillinam (J), 1 μg mL-1 avibactam (K), or the combination (L) or untreated (I). The strain KP7 (I-L) were treated for 6 hours before life-dead staining. 630x. .

Bocillin FL assay and SDS page

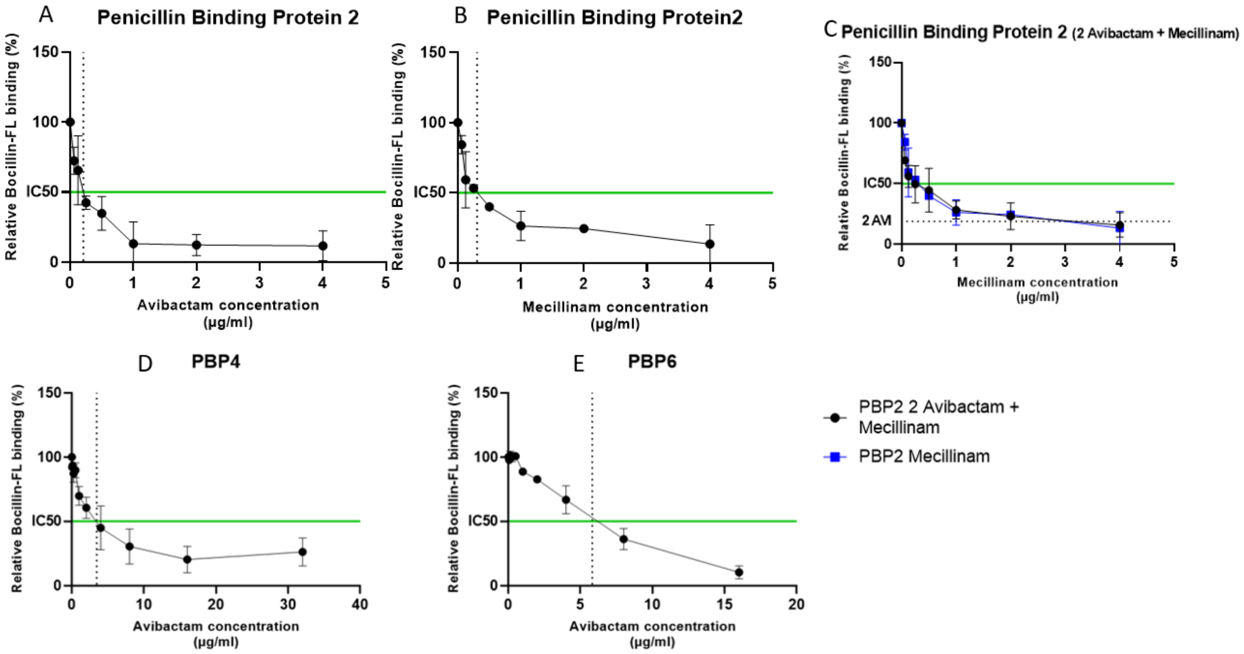

Figure S5. Fluorescence polarization (mP) of free Bocillin-FL at various concentration of avibactam and mecillinam. Increasing avibactam concentrations decreases the Relative Bocillin FL binding (%) to Penicillin Binding Protein2. Increasing mecillinam concentrations decreased Bocillin FL binding to Penicillin Binding Protein2. (C) Bocillin FL binding to Penicillin Binding Protein2 with fixed avibactam concentration at 2 µg/ml combined with increasing mecillinam concentration. 2 avibactam dotted line, average relative Bocillin Fl binding of four controls with only 2 µg/ml avibactam. Furthermore, increasing avibactam concentrations decreases the relative Bocillin FL binding (%) to (D) PBP4 and (E) PBP6.

Supplementary information on animal experiments

Mice were kept in quarantine/declamation from their arrival to the animal facility at age six weeks in Tecniplast T3 (12-90) cages with dedicated ventilation (Tecniplast, Italy), before the experiment started. During this time, they had unlimited access to food and water.

All experiments were carried out from 8 AM to 3 PM, in a climate-controlled class 2 animal facility at the University of Copenhagen's Animal test facility at the Faculty of Medical and Health Science. Mice were grouped randomly and kept divided into groups of 8 in each single-use plastic class 2 cage, Innovive. 4 mice for each treatment to each time point was decided upon based on a power calculation done on preliminary experiments done with the refence strains *E. coli* (ATCC25922) and *K. pneumoniae* (ATCC13883). With an Alpha level of 0.05. and a power of 90%, four mice in each group were optimal. During the whole experiment, all cages were ventilated through a dedicated filtered ventilation system. All mice had unlimited access to food and water during the whole experiment.

Clinical scoring according to the Danish Animal Ethical Council.

0: The mouse is unaffected.
1: The mouse is slightly affected (slightly slower movements or lightly stroked)
2: The mouse is affected (sits quietly but moves around when moving the crate, erected fur coat, slightly stiff belly, curved back, light toed).

3: The mouse is clearly affected (moves when pushed, possibly fur, half-closed eyes, sticky belly, curved back, toes). Should be sacrificed if it is judged that mice will reach score 4 before the next inspection.
4: The mouse is very affected (only moves reluctantly when it is pushed to, spiky fur coat, eyes closed, cold) shall be sacrificed.
5: The mouse is motionless and cold (lying on the side), shall be sacrificed.
6: The mouse is dead.

Table S3. ENA Accession numbers for raw Illumina reads for the study isolates.

Study ID	Strain ID	Bioproject	Accession no.	Source
KP1	CPO20150052	PRJEB36370	SAMEA6497202	Rectal swab
KP2	CPO20150004	PRJEB36370	SAMEA6497192	Urine
KP3	AMA0956	PRJEB50806	SAMEA12941027	Unknown
KP4	CPO20150068	PRJEB36370	SAMEA6497204	Rectal swab
KP5	CPO20170129	PRJEB36370	SAMEA6497258	Rectal swab
KP6	AMA0425	PRJEB50806	SAMEA12941025	Rectal swab
KP7	CPO20160073	PRJEB36370	SAMEA6497226	Rectal swab
KP8	CPO20140020	PRJEB36370	SAMEA6497186	Urine
KP9	CPO20150005	PRJEB36370	SAMEA6497193	Rectal swab
KP10	CPO20160030	PRJEB36370	SAMEA6497218	Urine
KP11	CPO20140036	PRJEB36370	SAMEA6497189	Pleural fluid
EC2	AMA0569	PRJEB50806	SAMEA12941026	Rectal swab
EC3	CPO20150010	PRJEB50806	SAMEA12941028	Groin swab
EC4	CPO20160003	PRJEB27363	SAMEA4744033	Urine
EC5	CPO20170042	PRJEB50806	SAMEA12941031	Urine
EC6	CPO20160050	PRJEB36710	SAMEA6533303	Urine
EC7	CPO20160043	PRJEB50806	SAMEA12941029	Urine
